# Infectious myocarditis: the role of the cardiac vasculature

**DOI:** 10.1007/s10741-018-9688-x

**Published:** 2018-03-14

**Authors:** Linde Woudstra, Lynda J. M. Juffermans, Albert C. van Rossum, Hans W. M. Niessen, Paul A. J. Krijnen

**Affiliations:** 1 Department of Pathology, VU University Medical Center, (room 0E46), De Boelelaan 1117, 1081 HV Amsterdam, The Netherlands; 2 Institute for Cardiovascular Research (ICaR-VU), VU University Medical Center, Amsterdam, The Netherlands; 3 Department of Cardiology, VU University Medical Center, Amsterdam, The Netherlands; 4 The Netherlands Heart Institute, Utrecht, The Netherlands; 5 Department of Cardiothoracic Surgery, VU University Medical Center, Amsterdam, The Netherlands

**Keywords:** Myocarditis, Infection, Vasculature, Endothelial cells, Myocardial infarction

## Abstract

Infectious myocarditis is the result of an immune response to a microbial infection of the heart. The blood vessels of the heart, both the intramyocardial microvasculature and the large epicardial coronary arteries, play an important role in the pathogenesis of infectious myocarditis. First of all, in addition to cardiomyocytes, endothelial cells of the cardiac (micro)vasculature are direct targets for infection. Moreover, through the expression of adhesion molecules and antigen presenting Major Histocompatibility Complex molecules, the blood vessels assist in shaping the cellular immune response in infectious myocarditis. In addition, damage and dysfunction of the cardiac (micro)vasculature are associated with thrombus formation as well as aberrant regulation of vascular tone including coronary vasospasm. These in turn can cause cardiac perfusion abnormalities and even myocardial infarction. In this review, we will discuss the role of the cardiac (micro)vasculature in the pathogenesis of infectious myocarditis.

**Electronic supplementary material:**

The online version of this article (10.1007/s10741-018-9688-x) contains supplementary material, which is available to authorized users.

## Introduction

Myocarditis is an inflammatory disease of the heart that is characterized by a large diversity in symptoms varying from a symptomless course to shortness of breath and mild flu-like symptoms, chest pain, specific or a specific ECG changes, to acute heart failure and chronically to dilated cardiomyopathy. [1] In the heart, myocarditis can induce cell loss, interstitial and replacement fibrosis, wall motion abnormalities, decreased ejection fraction, and arrhythmias. [2] Moreover, myocarditis is one of the leading causes of sudden cardiac death in young adults. [3] The cause of myocarditis can among others be an allergic or toxic reaction to medicines and toxic drugs as well as autoimmune organ-specific myocarditis and systemic autoimmune diseases-associated myocarditis. However, most often, the cause of myocarditis is an infection, including viruses, bacteria, protozoa, and fungi. In the Western world, viral infection is the most common cause of myocarditis. [4] At present, over 20 different viruses have been associated with viral myocarditis. [4] In South and Central America, infection of the protozoa *Trypanosoma cruzi* (*T. cruzi*) that leads to the so-called Chagas disease is the most prominent cause of myocarditis. [4, 5] Therefore, in this review, we will focus on viral- and *T. cruzi*-induced myocarditis. Of note, lymphocytic myocarditis refers to cases of myocarditis with the (immuno)histological appearance of intramyocardial foci of mainly infiltrated lymphocytes, that is generally considered to be most likely of a viral cause, but were no molecular detection was preformed to prove viral presence.

### Pathogenesis of viral-induced myocarditis

Most of our knowledge on the pathogenesis of viral myocarditis is obtained from studies in mice with experimental viral myocarditis. As such, we know that the pathophysiological course of viral myocarditis can be subdivided in three subsequent phases: an acute, subacute, and chronic phase. [6, 7] During the acute phase in the first days after infection, viral replication occurs within organs, including the heart. [6] In the subacute phase, approximately a week after infection, an immune response is activated in the heart aimed at clearing the virus resulting in increased levels of cytokines and immune cell infiltration into the myocardium. [8] In the chronic phase, 2 weeks after infection, the virus is usually cleared, myocardial inflammation resolved, and remodeling of the myocardium occurs. The damage of the heart in viral myocarditis can be the result of direct virus-related cardiac damage, i.e., the virus infects cardiac cells and kills them, and of autoimmunity, i.e., through mechanisms such as molecular mimicry the activated immune system also attacks the heart. [8] It has to be noticed that in part of the patients with viral myocarditis inflammation in the heart does not subside, but becomes chronic. This may be related to persistent viral infection in the heart or to an ongoing autoimmune response and can lead to the development of dilated cardiomyopathy.

### Pathogenesis of *T. cruzi*-induced myocarditis

The pathophysiological course of Chagas disease can be divided into an acute and chronic phase. The acute phase occurs 4–8 weeks after infection wherein *T. cruzi* spreads systemically into the heart and other organs. [5, 9] In the heart inflammation, necrosis of cardiomyocytes and amastigote nests of *T. cruzi* can be observed. [5] In case the parasite is not eliminated, the chronic phase of Chagas disease will develop in part of the patients. The chronic phase develops months, but usually decades after the primary infection. [10] The heart then is most commonly affected resulting in ventricular enlargement, thinning of the walls, interstitial fibrosis, mural thrombi, and arrhythmias. [11]

The blood vessels of the heart, both the intramyocardial microvasculature as well as the epicardial coronary arteries, play an important role in the pathogenesis of infectious myocarditis. They provide a barrier function to prevent blood-borne pathogens of entering the heart, but at the same time may form a prime target of infection. Moreover, they are a vital part of the post-infection immune responses in the heart. Furthermore, part of the clinical symptoms of patients with viral myocarditis or Chagas disease point to damage or dysfunction of the intramyocardial and/or epicardial blood vessels, such as coagulopathy, perfusion defects, and coronary spasms. In this review, we will discuss the structural and functional changes of the cardiac vasculature that occur with infectious myocarditis, caused by viral or *T. cruzi* infection, in more detail.

## Effects on the endothelium

### Infection of the cardiac (micro)vasculature

In infectious myocarditis, microbes such as cardiotropic viruses and *T. cruzi* infect the heart. However, of many myocarditis-associated viruses, detailed knowledge on which cell types are specifically infected in human infectious myocarditis remains scarce. In patients, viruses are usually identified via the detection of viral genome using polymerase chain reaction, which does not reveal the identity of the specific cells infected. [4] The loss of cardiomyocytes that occurs in infectious myocarditis suggests that the responsible microbes do infect cardiomyocytes. However, especially autopsy studies point out that there is a large variety in the amount of cardiomyocyte death in infectious myocarditis, which may be very limited, or even totally absent in case of borderline myocarditis. In addition, since autoimmunity can be triggered in infectious myocarditis, (part of the) damage to cardiomyocytes may also be the result of autoimmune responses. [12]

Another target of infection is the cardiac vasculature. To infect the heart, the endothelium of the cardiac microvasculature is the first barrier blood-borne infectious agents encounter. Of *T. cruzi* and more than 20 viruses that are associated with myocarditis, it is known that they can infect human and/or animal endothelial cells (Supplementary Table 1). In human autopsy material with proven myocarditis, it was demonstrated that Coxsackievirus, Cytomegalovirus, Dengue virus, Hanta virus, Herpes simplex virus, Parvovirus B19, and *T. cruzi* can infect the microvascular endothelial cells of the heart. [13–19] Also in animals, models with myocarditis, encephalomyocarditis virus, herpes simplex virus, and *T. cruzi* have been observed to infect cardiac endothelial cells. [20–23] Additionally, other cardiotropic viruses that are associated with myocarditis can infect cardiac endothelial cells in vivo and/or in vitro, of which is unknown whether they can infect the cardiac endothelial cells simultaneously with myocarditis. Lastly, there is an abundant number of cardiotropic viruses that are able to infect non-cardiac endothelial cells in vivo and/or in vitro that may potentially also infect cardiac endothelial cells (Supplementary Table 1).

The infection of the cardiac endothelium can cause among others endothelial activation, damage, and permeability. For instance, infection of cardiac endothelial cells in patients with viral myocarditis has shown to induce endothelial microparticles reflecting endothelial damage. [24] However, changes to the cardiac (micro)vasculature observed in infectious myocarditis may also be caused by non-viral mechanisms and that the exact cause of such changes, when encountered in vivo, is not known.

### Expression of adhesion molecules on the cardiac (micro)vascular endothelium

**Fig. 1:**
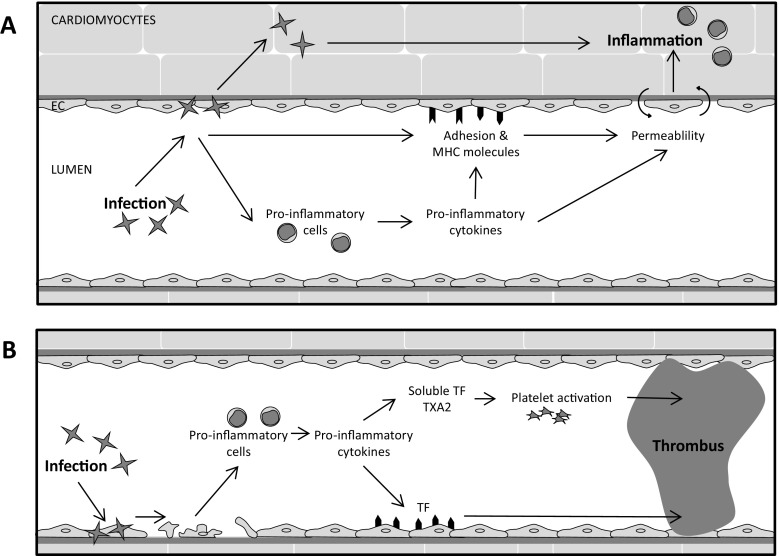
Schematic overview of the effect of infectious myocarditis in cardiac microvasculature on **a** the endothelial cells and **b** the coagulation. *EC* endothelial cells, *MHC* major histocompatibility complex, *TF* tissue factor, *TXA2* thromboxane A2

Infection of the heart and the subsequent death of cardiac cells induce an inflammatory response via the release of cytokines and chemokines that result in the expression of adhesion molecules at the site of infection and injury (schematically represented in Fig. 1a). It has been shown in endomyocardial biopsies (EMB) of patients with lymphocytic myocarditis and of patients with chronic Chagas disease that the expressions of Intercellular Adhesion Molecule 1 (ICAM-1), Vascular Cell Adhesion Molecule 1 (VCAM-1), and E-selectin on the capillary endothelium are elevated. [25–27] In the EMB of patients with lymphocytic myocarditis, respectively 81 and 46% of the cardiac blood vessels were positive for ICAM-1 or VCAM-1, whereas in control tissue obtained from patients with tetralogy of Fallot, 24% of the cardiac blood vessels was positive for ICAM-1 and 10% for VCAM-1. [25] Moreover, VCAM-1 was predominantly expressed on the endothelial cells of venules at the inflammatory sites in the EMB of part of the chronic Chagas patients. Similar, also mice with *T. cruzi* infection express significantly more VCAM-1 and ICAM-1 on their cardiac endothelium compared to mice without infection. [28]

This increase in endothelial adhesion molecule expression in the myocardium of patients with infectious myocarditis probably is related to pro-inflammatory cytokines, as elevated levels of the pro-inflammatory cytokines tumor necrosis factor-α (TNFα) and interleukin-1 (IL-1β) were shown to increase the expression of adhesion molecules ICAM-1, VCAM-1, and E-selectin on the endothelial cells of the human coronary artery and cardiac microvasculature. [29] Indeed, in part of the patients with viral-proven myocarditis or Chagas disease, elevated levels of pro-inflammatory cytokines have been measured. [30] Moreover, at autopsy, we observed accumulation of the advanced glycation end product (AGE) N^ε^-(carboxyethyl)lysine (CML) on the endothelium of intramyocardial blood vessels in patients with lymphocytic myocarditis. [27] It is known that AGEs such as CML can induce the expression of adhesion molecules on endothelial cells via binding to scavenger receptors such as the receptor for AGEs (RAGE) [31], and that RAGE expression is increased in the hearts of mice after Coxsackievirus B3-induced acute myocarditis [32].

On the one hand, the presence of pathogens themselves can affect endothelial adhesion molecule expression. Indeed, in vitro studies have shown upregulation of ICAM-1 and VCAM-1 expression in human endothelial cells upon infection with myocarditis-associated viruses (adenovirus, arenavirus, coxsackievirus B3 (CVB3), cytomegalovirus, and West Nile virus) or upon infection with *T. cruzi*. [33, 34] Interestingly, many viruses including rhinovirus, coxsackieviruses, picorna virus, and encephalomyocarditis virus utilize adhesion molecules such as ICAM-1 and VCAM-1 as cell recognition and cell entry receptors. [35, 36] Thus, the virus-induced adhesion molecule upregulation may provide a positive feedback and exacerbate infection.

On the other hand, adhesion molecules on cardiac microvascular endothelial cells control leukocyte traffic between the blood and the myocardium. Therefore, elevated expression levels of adhesion molecules also increases the infiltration rate of inflammatory cells into the affected myocardium in patients with myocarditis, thereby facilitating the clearance of virus and virus-infected cells. However, chronically increased endothelial adhesion molecule expression in the cardiac microvasculature, whether or not through persistent viral infection [33], may facilitate autoimmune responses in the heart and the development of chronic heart failure.

### Expression of major histocompatibility complex molecules on the cardiac (micro)vascular endothelium

Another set of cell surface proteins that are important in shaping the immune response in the heart in infectious myocarditis are the major histocompatibility complex (MHC) molecules or their human variant the Human Leucocyte Antigen (HLA). MHC molecules display processed self and non-self-antigens on the cell surface for the specific recognition by T cells for appropriate action in case of infection-related non-self-antigen. MHC class I molecules are expressed on all nucleated cells, including cardiac endothelial cells, whereas the expression of MHC class II molecules is usually restricted to professional antigen presenting cells.

In EMB of patients with lymphocytic or viral myocarditis and of patients with chronic Chagas disease, increased expression of MHC class I but also MHC class II molecules was observed on the endothelium of the cardiac microvasculature (Fig. 1a). [37–40] It seems likely that the cardiac microvascular endothelial cells use these MHC molecules to display antigens of the infectious agent to effector or helper T cells to control the infection. MHC class I interacts with CD8+ cytotoxic T cells that can mediate destruction of the infected cells, whereas MHC class II interacts with CD4+ helper T cells initiating a specific immunity. In addition, the control of several cardiotropic viruses, including parvovirus B19 [41], influenza [42], dengue [43], hepatitis B virus [44], and herpesviruses [45] may depend significantly on MHC class II-activated cytotoxic CD4+ effectors.

The increased expression of endothelial MHC molecules can be induced by interferon gamma (IFN-γ), a cytokine that is released commonly during virus infections. [46] Indeed, INFγ levels were shown to be increased in both mice viral and *T. cruzi*-induced myocarditis. [47, 48] Alternatively, the induction of MHC molecules might be directly virus related as both MHC class I and II expression was shown to be induced in endothelial cells in vitro by West Nile virus infection. [34]

The expression of MHC class II on the cardiac endothelium may also relate to the development of autoimmunity and chronic myocarditis after infectious myocarditis [49], although the precise mechanisms are not yet clear. On the one hand it was shown in mice that absence of non-hematopoietic MHC class II expression prevented the development of experimental autoimmune myocarditis [49], suggesting that the presence of MHC class II on cardiac cells elicits autoimmunity development. Moreover, the susceptibility to develop autoimmunity may be related to certain HLA alleles as the introduction of human HLA-DQ8 alleles in non-obese diabetic (NOD) mice induced spontaneous autoimmune myocarditis and dilated cardiomyopathy [50], which underscores the possibility of a genetic MHC class II-related predisposition in patients who develop chronic myocarditis and dilated cardiomyopathy after infectious myocarditis. Conversely, mice lacking MHC class II were more susceptible to long-term cardiac injury and viral persistence after Coxsackievirus B3 infection, suggesting that MHC class II-mediated immune responses are necessary to prevent chronic myocarditis. [51]

### Increased permeability of the cardiac (micro)vasculature

In patients with myocarditis, an increase in intramyocardial edema is a frequently observed phenomenon during tissue characterization by cardiac magnetic resonance (CMR). [1, 52] This increase in edema points to an increased permeability of the cardiac microvasculature in patients with infectious myocarditis. In addition, increased permeability has been observed in the epicardial coronary arteries of patients with lymphocytic myocarditis. [53]

Multiple causes may underlie this increase in vascular permeability in infectious myocarditis (Fig. 1a). An increase in vascular permeability normally accompanies inflammatory responses in the underlying tissue. For instance, the elevated levels of pro-inflammatory cytokines that are present in the inflamed heart can affect the permeability of the (micro)vasculature. Also, the sometimes extensive recruitment and extravasation of immune cells into the myocardium may increase the cardiac (micro)vascular permeability. [54] Moreover, accumulation of the aforementioned AGE CML in the cardiac microvasculature may associate with increased permeability as was shown for diabetic vasculopathy hamsters. [55]

Alternatively, infection of endothelial cells, that can occur in infectious myocarditis (Supplementary Table 1), has been shown to affect vascular permeability directly. Its barrier function might decrease through infection-induced death of endothelial cells. For instance, infection of cardiac microvascular endothelial cells with Coxsackievirus B3 resulted in an increase of apoptosis-related caspase-3 activity and Bax/Bcl-2 protein ratio, coinciding with an elevated microvascular permeability. [56] Moreover, viral infection in endothelial cells was shown to induce decreased levels of VE-cadherin [57], enlarged intercellular gap junctions [56], and disruption of the tight junctions [58]. Conversely though, dengue virus infection of human endothelial cells in vitro led to an improved barrier function [59], suggesting that the mechanisms that underlie infection-induced (micro)vascular permeability may differ between infectious agents. In theory, the increased vascular permeability and loss of barrier function would facilitate the infection of the underlying tissue, as was for instance shown to occur in mice infected with *T. cruzi*. [60]

### Proliferation of the (micro)vascular cells

Infectious myocarditis may also induce structural changes of the cardiac vasculature. In rats with *T. cruzi*-induced acute Chagas disease, an increase of both volume and length of blood vessels was shown within the ventricles and atria of the heart. [61] Little is known about the structural cardiac vascular changes in patients, although in patients with chronic Chagas disease increased arteriole and capillary diameters were observed within the ventricles. [62] A possible explanation for these alterations of the cardiac microvasculature is that infectious myocarditis induces proliferation of endothelial cells. In mice with CVB3-induced myocarditis, an increase in the number of proliferating endothelial cells was found within the myocardium, which increased 3 days after infection, peaked at day 7, and finally decreased at day 21. [63] Unclear is whether this increase in proliferating endothelial cells in this study is associated with angiogenesis or re-epithelization. [63] Nevertheless, in mice with CVB3-, CVB4-, and encephalomyocarditis virus-induced myocarditis, narrowed capillary lumens have been observed [64, 65], which may be an explanation for the observed endothelial cell proliferation. Next to endothelial cell proliferation, *T. cruzi* infection has also been shown to stimulate proliferation of vascular smooth muscle cells in vitro. [66]

## Induction of thrombosis

The putative infection, pro-inflammatory activation, and death of cardiac endothelial cells each create a potential procoagulant environment. Indeed, occlusive thrombi, fibrin deposits, and aggregated platelets have been found in the small epicardial and intramyocardial vasculature of *T. cruzi*-infected mice and dogs and in mice with CVB3-induced myocarditis. [67, 68] Such microvascular occlusions could provoke local ischemia in the myocardium as has been suggested to occur in Chagas myocarditis. [69] In addition, mural thrombi (i.e., thrombi that formed on the endocardium) have been found in patients with Chagas disease or lymphocytic myocarditis (Fig. 1b). [70, 71] Similarly, mural thrombi have been observed in mice with CBV3-, encephalomyocarditis virus-, or *T. cruzi-*induced myocarditis. [68, 72–74] Moreover, in mice with CVB3-induced myocarditis, the chance of mural thrombi increased over time. [73] Such mural thrombi pose a clear danger for thromboembolism.

In the hearts of mice with CVB3-induced myocarditis and in EMB of patients with suspected inflammatory cardiomyopathy, an increase in tissue factor (TF) expression was shown. [72] TF is the key initiator of the extrinsic pathway of coagulation. [75] Cardiac TF normally is expressed on cardiomyocytes and is important for maintaining homeostasis in the heart and prevents against spontaneous cardiac hemorrhage. [76] However, in mice with CVB3-induced myocarditis, TF was found to be expressed also on the surface of the cardiac microvasculature, coinciding with fibrin deposits [72], underscoring that the luminal surface of the cardiac microvasculature can become intently procoagulant in infectious myocarditis. Nevertheless, whether cardiac microvascular TF expression occurs on endothelial cells or on subendothelial tissue that becomes exposed after endothelial damage, or both, is not certain yet. Cardiac microvascular endothelial TF expression has been observed in the hearts of patients with myocardial infarction [77], indicating that cardiac microvascular endothelial cells can express TF. Moreover, infection of human umbilical vein endothelial cells with myocarditis-associated viruses in vitro has been shown to increase TF expression and blood clotting. [78, 79] In addition, pro-inflammatory cytokines such as TNFα and IL6 were shown to increase the expression of TF as well as soluble TF in human umbilical vein endothelial cells. [72, 80]

Increased thrombogenicity has also been observed in the blood in infectious myocarditis. In mice with CVB3 myocarditis, increased TF activity was found in the blood [72], and in patients with chronic Chagas disease increased levels of soluble TF and an increased endogenous thrombin potential in the blood have been observed (Fig. 1b). [81] Moreover, platelets isolated from mice infected with *T. cruzi* were more prone to aggregate than those from uninfected controls. [82] This may be related to an increased production of the eicosanoid thromboxane A2 (TXA2), by platelets and/or endothelial cells, which can amplify platelet activation via autocrine or paracrine pathways. [82, 83] Also, in mice with Chagas disease, elevated levels of TXA2 are found in the plasma. [82, 84] The prothrombotic state may be caused through the damage of the endothelial cells as in rats with acute Chagas disease there is swelling of the endothelial cells and endothelium damage through exposing the subendothelial collagen. [85]

Importantly, the hypercoagulable state that exists in infectious myocarditis appears to be a double-edged sword. On the one hand, excessive clotting can lead to intravascular coagulation and subsequent tissue damage [86], while on the other hand activation of coagulation during infection is a protective mechanism to limit the spread of infectious pathogens. [6] Coagulation proteases, such as thrombin, can activate cells by cleavage of protease-activated receptors (PARs), which can modulate the immune response to a viral infection. [86] In addition, the expression of PARs is increased in endothelial cells after cardiotropic viral infection. [87] In PAR-1-deficient mice, infection with CVB3 led to increased viral loads in the heart and increased cardiac injury compared with wild-type mice [88], pointing to the importance of the TF/thrombin/PAR pathway in anti-viral immunity. Conversely, however, PAR-2-deficient mice had a reduced viral load in the heart and were protected from developing myocarditis. [89] These studies highlight that PARs modulate the innate immune response and can have both positive and negative effects identify that the PAR receptors and thereby the coagulation system plays an important role in the immune response in viral induced myocarditis.

## Effects on coronary artery atherosclerosis

Aberrations in the blood vessels of the heart in infectious myocarditis are not limited to the intramyocardial microvasculature but can also occur in the large epicardial coronary arteries and in atherosclerotic plaques therein. [90] A substantial proportion of infectious myocarditis patients have complaints of chest pain with angina-like characteristics that point to coronary artery disease or even myocardial infarction. [91–93] Infectious myocarditis patients can present with additional infarct-like symptoms such as ST-segment elevation on ECG, wall motion abnormalities, and elevated circulating cardiac troponin levels. [94, 95] However, despite the similarities in clinical presentation, infectious myocarditis and myocardial infarction are generally considered as distinct clinical entities.

**Fig. 2:**
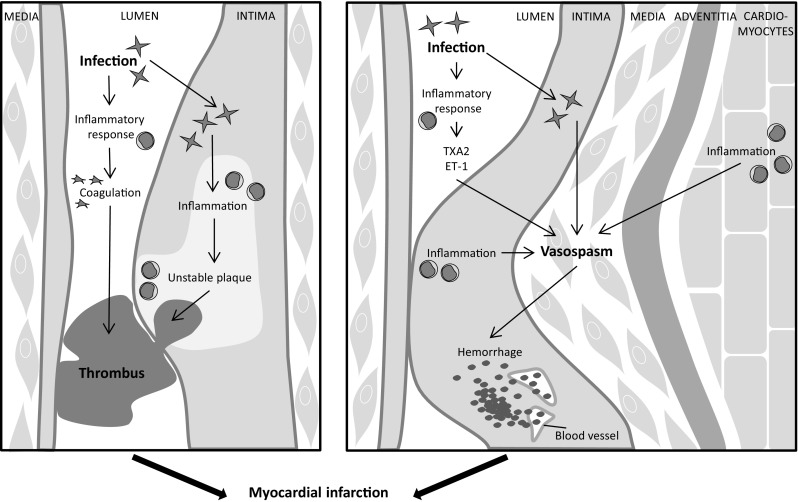
Schematic overview of the effect of infectious myocarditis on epicardial coronary arteries that may induce myocardial infarction. *ET-1* endothelin-1, *TF* tissue factor, *TXA2* thromboxane A2

In clinical practice, infectious myocarditis is only considered as the underlying cause of infarct-like complaints when myocardial infarction is ruled out, based on the absence of angiographic coronary narrowing or obstruction. In case of normal or non-obstructed coronary arteries, use of CMR imaging has been suggested as a complementary imaging tool to further discriminate between myocarditis and myocardial infarction, wherein myocardial injury is mainly located in the subendocardium with myocardial infarction as opposed to a more (sub)epicardial location with myocarditis. [96, 97] Indeed, in 50 to 78% of the patients with acute chest pain but without angiographically determined obstructed coronary arteries, the underlying cause was found to be acute myocarditis. [93, 98, 99] Several observations suggest an interrelatedness between infectious myocarditis and myocardial infarction. Recent respiratory tract influenza virus infections for instance, a virus commonly associated with myocarditis, are significantly associated with the development of myocardial infarction also [100], while vaccination against influenza is associated with a decreased risk of myocardial infarction [101]. In addition, enteroviruses (Coxsackie B viruses) were detected in the hearts of 40% of patients who died of sudden myocardial infarction versus only 4% of matched subjects without cardiac disease. [102] 103] Moreover, one case report demonstrated the co-occurrence of EMB-confirmed parvovirus B19-related myocarditis and acute myocardial infarction caused by a thrombus-occluded epicardial coronary artery (Fig. 2). [104] Another case with myocardial infarction due to coronary thrombosis was reported, wherein enterovirus was detected postmortem both in the coronary atherosclerotic plaque and in heart tissue. [105] Similarly, in a third case, presumed myopericarditis co-occurred with coronary angiography and CMR proven acute posterior myocardial infarction, related to a right coronary artery thrombosis, and a concurrent Coxsackievirus B2 infection. [106] Furthermore, we have recently shown in autopsied cases of patients diagnosed postmortem with lymphocytic myocarditis a high prevalence of very recent myocardial infarction, some with thrombus-occluded coronary arteries. [90] These data demonstrate that infectious myocarditis and myocardial infarction can be present simultaneously, also in patients with occluded epicardial coronary arteries. Even more, these data suggest that infectious myocarditis either via a direct virus effect or indirectly via the activated immune response and inflammation can facilitate the development of myocardial infarction.

Myocardial infarction most often is the result of coronary atherosclerotic plaque complication, either through rupture or erosion of plaques, followed by thrombus formation. Intraplaque inflammation is an important mediator of atherosclerotic plaque destabilization that renders them more vulnerable for complication. [107] We observed increased intraplaque infiltration of macrophages and neutrophils, increased intraplaque hemorrhage, and increased instability in coronary artery atherosclerotic plaques of autopsied lymphocytic myocarditis patients [90] (Fig. 2), indicating that infectious myocarditis may facilitate myocardial infarction through an effect on atherosclerotic plaques.

In theory, these effects could be the result of direct infection of coronary artery plaques. As many cardiotropic viruses have been shown to infect endothelial cells (Supplementary Table 1), they may also do so in the epicardial coronary arteries and coronary atherosclerotic plaques [108–112]. In fact, a wide range of pathogens, including cardiotropic viruses, have been identified by genome or antigen detection methods in human atherosclerotic plaques [108], although, to the best of our knowledge, infection of atherosclerotic lesions with *T. cruzi* has never been reported. The cardiotropic viruses cytomegalovirus [109–111], herpes simplex virus [110] enterovirus (echovirus 9 and coxsackieviruses B1 and B3) [109], Epstein-Barr virus [110], and parvovirus B19 [112] have been found in human coronary atherosclerotic plaques. In fact, enhanced cytomegalovirus infection was found in human atherosclerotic vessels compared to non-atherosclerotic vessels [111], suggesting that atherosclerotic plaques have increased susceptibility for viral infection. However, it has to be noticed that in these studies concomitant myocarditis was not reported. Although the precise mechanisms are not exactly known, it has become clear from studies in animal models that viral infection can induce or accelerate atherosclerosis. In 1978, it was shown in chickens that infection with Marek’s disease herpesvirus, either or not in combination with a high fat diet, resulted in grossly visible atherosclerotic lesions in large coronary arteries and aortas, while these were absent in uninfected chickens. [113] Moreover, in atherosclerotic ApoE^−/−^ mice, influenza A has been reported to directly infect aortic atherosclerotic lesions, as well as the heart. [114] This resulted in higher levels of chemokines and cytokines in the blood and the plaque as well as a higher density of intraplaque macrophages. Interestingly, intraplaque inflammation and thrombotic effects were also observed in ApoE^−/−^ mice infected with influenza A, but without evidence of direct infection of the plaques. [115]. It thus seems that infection with influenza, or other cardiotropic viruses, might enhance the chance of a myocardial infarction through increased plaque inflammation and resultant plaque complication, whether or not through direct plaque infection, via increased inflammation in the blood or in the heart and a prothrombotic state. [101] However, such mechanism remains to be proven.

Alternatively, the cardiac and systemic inflammation associated with infectious myocarditis may by itself affect atherosclerotic plaques, irrespective of the infectious pathogens. Indeed, studies have shown that different diseases characterized by increased systemic or cardiac inflammation, including rheumatoid arthritis, vasculitis, and myocardial infarction itself, can accelerate atherosclerosis. [116–118] However, limited studies of the context of atherosclerosis are known in patients with Chagas disease. Nonetheless, microscopy revealed that patients with chronic Chagas disease had morphological similar atherosclerosis as patients without Chagas disease. [119] Moreover, patients with and without Chagas disease presenting a myocardial infarction had a comparable frequency of angiographically normal coronary arteries. [120] Nevertheless, there are a few reports that do show that Chagas disease can coincide with myocardial infarction. [121–123]

## Effects on coronary vasomotor function

Patients with myocarditis frequently report with chest pain complaints, often in the absence of significant coronary obstructive artery disease. [124, 125] These angina pectoris-like symptoms are likely caused by an aberrant vasomotor function of the coronary vasculature. This may be the result of reduced vasodilator capacity and/or hyper-constriction (spasm) of coronary (micro)vasculature. Coronary artery spasm has been frequently reported in patients with viral myocarditis (Fig. 2). [126–128] Already in 1985 vascular spasm in the arterioles and capillaries in mice with Chagas disease was reported. [129]

An often used technique to determine whether coronary arteries are sensitive to vasospasm is to expose the coronary arteries to acetylcholine, which can have a direct vasoconstricting action on the vascular smooth muscle provoking coronary vasospasm, and to analyze the vascular responses by angiography. [128] In addition, acetylcholine may also affect the vasodilating action mediated by the endothelium of the coronaries. [130] In patients with Chagas disease and patients with EMB-proven lymphocytic myocarditis, impaired endothelium-dependent impaired coronary vasodilation was demonstrated in the presence of acetylcholine, which may reflect abnormal endothelial function. [53, 131] Similarly, decreased acetylcholine vasodilation was also found in the coronary arteries of mice with CVB3-induced chronic myocarditis. [132]

The underlying cause of vasospasm in patients with infectious myocarditis may relate to presence of the infection itself. Of patients with inflammatory cardiomyopathy, it is known that the endothelial function is impaired in patients with virus persistence compared to patients without virus. [133] Moreover, the endothelial dysfunction was more pronounced in patients that showed increased inflammation in the myocardium. In addition, in patients with clinical myocardial infarction, but with angiographically normal coronary arteries, viral genome could be detected in 71% of the EMB, mainly parvovirus B19, but also enterovirus and adenovirus. [92] Likewise, in patients with clinically suspected myocarditis, exposing the coronary arteries to acetylcholine resulted in coronary vasospasm in 70.9%. [128] Of these patients, the ones with proven parvovirus B19 myocarditis had significantly more often coronary vasospasm than patients without myocardial inflammation and/or virus. [128] These studies suggest that virus infection can interfere with the endothelial function of the coronary arteries.

Furthermore, the initiation of vasospasm and/or endothelial dysfunction may also relate to the increased inflammation within the myocardium in patients with infectious myocarditis (Fig. 2). In patients with lymphocytic myocarditis, a correlation was noted between the number of lymphocytes in the myocardium and the epicardial coronary constriction in response to acetylcholine. [53] In patients with myocarditis also, a correlation between impaired endothelial function, determined by flow-mediated vasodilation in the peripheral radial arteries, and enhanced endothelial expression of MHC and adhesion molecules in EMB was observed. [132] Furthermore, coronary vasospasm was found to occur often at the site of atherosclerotic lesions which are associated with impaired endothelium-dependent vasodilation. [130, 134] These studies indeed suggest that coronary vasospasm is associated with inflammation in the heart.

The occurrence of coronary vasospasm in patients with infectious myocarditis may relate to vasoactive substances, like the powerful vasoconstrictors Endothelin-1 (ET-1) and TXA2 [69, 135] (Fig. 2). In humans with chronic Chagas disease, increased plasma levels of ET-1 have been demonstrated. [136] Similarly, also in mice with Chagas disease or viral myocarditis, elevated levels of ET-1 in the plasma have also been observed, but also increased expression of ET-1 on the endothelium of the coronary arteries. [137–139] Elevated levels of TXA2 in the plasma and increased gene expression of thromboxane synthase in the myocardium were demonstrated in mice with *T. cruzi-*induced Chagas disease. [80, 84] Interestingly, *T. cruzi* is also capable of synthesizing TXA2 itself and the majority of TXA2 in the blood of infected mice is derived from the parasite. [140]

We have observed in autopsied patients with lymphocytic myocarditis elevated intraplaque hemorrhage and mast cells in the epicardial coronary arteries. [90] A possible cause of intraplaque hemorrhage is coronary vasospasm. Interestingly, via the secretion of vasoactive factors such as histamine, chymase, and tryptase, mast cells have also been associated with coronary vasospasm. [141]

## Effects on cardiac perfusion

The above described prothrombotic, pro-atherosclerotic, and vasospastic effects can in theory result in impaired perfusion of the heart, resulting in (transient) cardiac ischemia. [8] One important parameter to assess whether the functional and structural abnormalities of the cardiac vessels interfere with the blood circulation is the coronary flow reserve, i.e., the degree of the increase in coronary flow from the basal to the maximal coronary vasodilation. Reduced coronary flow reserve indicates the presence of either a flow-limiting coronary artery stenosis and/or dysfunction of the coronary microcirculation. [142, 143] Impaired coronary flow reserve has been observed in patients with viral myocarditis and in patients with acute Chagas disease [144–148], but also patients with chronic Chagas disease [69, 147, 148]. The impaired coronary flow reserve in acute Chagas patients has been correlated with age, severity of Chagas, and ventricular heart function. [142, 146] This decreased cardiac perfusion in Chagas disease was observed in patients with angiographically normal coronary arteries, supporting the concept that abnormal flow regulation essentially occurs at the microvascular level. [142, 144–146] Similarly, in patients with viral myocarditis mimicking acute coronary syndrome with angiographically normal coronary arteries, a reduced coronary flow was observed up to 1 year after hospital administration compared to healthy participants. The coronary flow was especially reduced during the first week and recovered with time. [149]

The presence of virus in the heart may affect the cardiac perfusion, since in patients with EMB-proven myocarditis the coronary blood flow was found to be significantly impaired when myocardial virus persisted compared to absence of virus detection. [150] In mice with CVB3-induced myocarditis, the coronary flow reserve was significantly reduced 1 week after CVB3 infection and was still impaired 2 weeks after infection and correlated with the severity of myocarditis. [143] Moreover, in mice with encephalomyocarditis virus-induced myocarditis, a decreased perfusion of the small intramyocardial arteries has been observed. [151] Also, mice infected with *T. cruzi* had decreased microcirculatory flow within the arterioles and venules. The arterioles of these infected mice exhibited segmental areas of vasospasm and dilatation. [152]

## Conclusion

In this review, we have provided an overview of the functional and structural changes in the coronary (micro)vasculature that occur in infectious myocarditis and the effects that these changes have or may have on its pathogenesis. Clearly, the coronary (micro)vasculature plays a prominent role in the different stages of the disease; initially as a barrier against and as a target for infection, and subsequently as an important factor in the shaping of the immune response in the heart and as an important determinant of dysfunction of the heart. As such, these changes in the coronary (micro)vasculature may explain, in part, the wide variety of clinical symptoms in infectious myocarditis patients. Moreover, evidence is accumulating that infectious myocarditis can co-occur with myocardial infarction and that it may facilitate its development via destabilization of atherosclerotic plaques. Given that infectious myocarditis is believed to be clinically silent in most cases, it may be an undetected underlying cause in patients that present with myocardial infarction. However, more research is needed to determine the clinical and prognostic implications of disease co-occurrence.

## Electronic supplementary material

ESM 1 (DOCX 252 kb)
