# Supplementary material for: Infectious myocarditis: the role of the cardiac vasculature
Source: Heart Fail Rev. 2018 Mar 14;23(4):583–95. doi: 10.1007/s10741-018-9688-x (PMC6010496; doi:10.1007/s10741-018-9688-x)
Supplement: Supplementary file 1 — (DOCX 252 kb) [file 10741_2018_9688_MOESM1_ESM.docx]

**Infectious myocarditis: the role of the cardiac vasculature**

Journal: Heart failure reviews

Correspondence Linde Woudstra: VU University Medical Center, Department of Pathology (room 0^E^46), De Boelelaan 1117, 1081 HV Amsterdam, The Netherlands.

Phone: +31204443292; Fax: +31204442964; Email: [l.woudstra@vumc.nl](mailto:l.woudstra@vumc.nl).

Supplementary table 1. *T. cruzi* and viruses associated with myocarditis that infect endothelial cells.

| Infectious agent | **Cardiac endothelial cells** | **Non cardiac endothelial cells** |
| --- | --- | --- |
| Studies in humans with infectious myocarditis | | |
| Coxsackievirus | Autopsy: heart microvascular ECs[1, 2] | - |
| Cytomegalovirus | Autopsy: heart microvascular ECs[1] | - |
| Dengue virus | Autopsy: heart microvascular ECs[3, 4] | - |
| Hanta virus | Autopsy: heart microvascular ECs[5] | - |
| Herpes simplex virus | Autopsy: heart microvascular ECs[6] | - |
| Parvovirus B19 | Autopsy: cardiac microvascular ECs[7] | - |
| T. cruzi | Autopsy: heart microvascular ECs[8] | - |
| Studies in animals models of infectious myocarditis | | |
| Encephalo-myocarditis virus | *In vivo:* porcine cardiac microvascular ECs[9], mouse adventitial cells of coronary arteries, veins and capillaries[10] | - |
| Herpes simplex virus | Autopsy: equine heart microvascular ECs[11] | - |
| T. cruzi | *In vivo*: mice cardiac ECs[12] | - |
| Studies without diagnosed infectious myocarditis | | |
| Adenovirus | *In vitro*: human, rat and bovine aortic ECs[13-15] | *In vitro*: HUVECs[15-18]  *In vivo*: rat aortic ECs[13], rabbit corneal ECs[19], rat carotid arteries ECs[17] |
| Arbovirus | - | *In vitro*: mouse brain ECs[20] |
| Arenavirus virus | - | *In vitro*: HUVECs[21-23] |
| Chikungunya virus | - | *In vivo*: mouse vascular ECs[24] |
| Coxsackievirus | - | *In vitro*: bovine aortic ECs[15], bovine pulmonary artery ECs[25], bovine thoracic aorta ECs[25], bovine vena cava ECs[25], human brain microvascular ECs[26], human, pancreatic microvascular ECs[27], HUVECs[28-32] |
| Cytomegalovirus | *In vitro*: human coronary artery ECs[33], human and porcine heart microvascular ECs[34, 35], porcine vena cava microvascular ECs[35] | *In vitro*: human and porcine aortic ECs[35-37], human internal mammary artery ECs[33], human saphenous vein ECs[33], human adult iliac artery ECs[38], human placental microvasculature ECs[38], human corneal ECs[39], human sinusoidal ECs[40], human brain microvascular ECs[37], human dermal microvascular ECs[41], HUVECs[18, 38, 40, 42-47]  *In vivo:* human placental ECs[45]  Autopsy: human carotid plaques ECs[44] |
| Dengue virus | - | *In vitro*: human dermal microvascular ECs[48-50], mouse brain ECs[51], human pulmonary ECs[52], HUVECs[53-56] |
| Encephalo-myocarditis virus | *In vitro*: mouse cardiac vascular ECs[57] | *In vivo*: mouse brain microvascular ECs[58], mouse spinal cord microvascular ECs[58], mouse funiculus lateralis ECs[59] |
| Epstein-Barr virus | - | *In vitro*: HUVECs[60, 61] |
| Hanta virus | - | *In vitro:* bovine aortic ECs[62], human saphenous veins ECs[63], human lung microvascular ECs[64, 65], HUVECs[66-69] |
| Hepatitis C | - | *In vitro*: human dermal microvascular ECs[70], human brain microvascular ECs[71], human liver sinusoidal ECs[71], HUVECs[70-73]  *In vivo:* human tubular capillary ECs[74] |
| Herpes simplex virus | *In vitro*: human heart microvascular ECs[75] | *In vitro*: bovine aortic ECs[15], human aortic ECs[75, 76], bovine pulmonary artery ECs[25], bovine thoracic aorta ECs[25], bovine vena cava ECs[25], rat cerebral microvascular ECs[77], human corneal ECs[78-80], human dermal microvascular ECs[81], mouse brain microvascular ECs[82], HUVECs[15, 42, 75, 83-85]  *In vivo*: human corneal ECs[86]  Autopsy: human brain microvascular ECs[87] |
| Human immunodeficiency virus | - | *In vitro*: human brain microvascular ECs[88], human adipose ECs[89], HUVECs[90] |
| Influenza virus | - | *In vitro*: bovine aortic ECs[15], human lung microvascular ECs[91-94], HUVECs[15, 18, 95, 96]  *In vivo:* mouse cerebral ECs[97] |
| Measles virus | - | *In vitro*: bovine aortic ECs[15], bovine pulmonary artery ECs[25], bovine thoracic aorta ECs[25], bovine vena cava ECs[25], human and mouse brain microvascular ECs[82, 98, 99], human cerebral ECs[100], HUVECs[15, 18, 42, 84, 99]  *In vivo*: mouse brain ECs[98]  Autopsy: human brain vascular ECs[101, 102] |
| Mumps virus | - | *In vitro*: bovine pulmonary artery ECs[25], bovine thoracic aorta ECs[25], bovine vena cava ECs[25], HUVECs[15] |
| Parvovirus B19 | *In vitro*: human cardiac microvascular ECs[103, 104], human coronary artery ECs[105] | *In vitro*: human aortic ECs [105], human placenta microvascular ECs[106], human dermal microvascular ECs [105], human pulmonary artery ECs[105], HUVECs [105, 107]  *In vivo:* human skin vascular ECs[108, 109] |
| Polio virus | - | *In vitro*: human brain microvascular ECs[26], HUVECs[15, 29] |
| Respiratory syncytial virus | - | *In vitro*: human pulmonary microvascular ECs[110], HUVECs[18] |
| Rubella virus | - | *In vitro*: human saphenous vein ECs[111], HUVECs[111, 112]  Autopsy: human placenta ECs[113] |
| T. cruzi | - | *In vitro*: human coronary artery ECs[114], HUVECs [12, 115-119] |
| Vaccinia virus | - | *In vitro*: HUVECs[120] |
| Varicella virus | - | *In vivo:* human dermal ECs[121]  Autopsy: human cerebral and temporal arteries ECs[122] |
| West Nile virus | - | *In vitro:* human brain microvascular ECs[123-125], HUVECs[46, 126]  Autopsy: human dermal microvascular ECs[127], human pulmonary microvascular ECs[127] |
| Yellow fever | - | *In vitro*: HUVECs[128] |

ECs: endothelial cells, HUVECs: human umbilical vein endothelial cells.

**References**

[1] Iwasaki T, Monma N, Satodate R, Segawa I, Oyama K, Kawana R, et al. Myocardial lesions by Coxsackie virus B3 and cytomegalovirus infection in infants. Heart Vessels Suppl. 1985;1:167-72.

[2] Deguchi H, Fujioka S, Terasaki F, Ukimura A, Hirasawa M, Kintaka T, et al. Enterovirus RNA replication in cases of dilated cardiomyopathy: light microscopic in situ hybridization and virological analyses of myocardial specimens obtained at partial left ventriculectomy. J Card Surg. 2001;16:64-71.

[3] Salgado DM, Eltit JM, Mansfield K, Panqueba C, Castro D, Vega MR, et al. Heart and skeletal muscle are targets of dengue virus infection. Pediatr Infect Dis J. 2010;29:238-42.

[4] Povoa TF, Alves AM, Oliveira CA, Nuovo GJ, Chagas VL, Paes MV. The pathology of severe dengue in multiple organs of human fatal cases: histopathology, ultrastructure and virus replication. PLoS One. 2014;9:e83386.

[5] Saggioro FP, Rossi MA, Duarte MI, Martin CC, Alves VA, Moreli ML, et al. Hantavirus infection induces a typical myocarditis that may be responsible for myocardial depression and shock in hantavirus pulmonary syndrome. J Infect Dis. 2007;195:1541-9.

[6] Leveque N, Boulagnon C, Brasselet C, Lesaffre F, Boutolleau D, Metz D, et al. A fatal case of Human Herpesvirus 6 chronic myocarditis in an immunocompetent adult. J Clin Virol. 2011;52:142-5.

[7] Bultmann BD, Klingel K, Sotlar K, Bock CT, Baba HA, Sauter M, et al. Fatal parvovirus B19-associated myocarditis clinically mimicking ischemic heart disease: an endothelial cell-mediated disease. Hum Pathol. 2003;34:92-5.

[8] Higuchi Mde L, De Brito T, Martins Reis M, Barbosa A, Bellotti G, Pereira-Barreto AC, et al. Correlation between Trypanosoma cruzi parasitism and myocardial inflammatory infiltrate in human chronic chagasic myocarditis: Light microscopy and immunohistochemical findings. Cardiovasc Pathol. 1993;2:101-6.

[9] Vlemmas J, Billinis C, Psychas V, Papaioannou N, Paschaleri-Papadopoulou E, Leontides S, et al. Immunohistochemical detection of encephalomyocarditis virus (EMCV) antigen in the heart of experimentally infected piglets. J Comp Pathol. 2000;122:235-40.

[10] Burch GE, Rayburn P. EMC viral infection of the coronary blood vessels in newborn mice: viral vasculitis. Br J Exp Pathol. 1977;58:565-71.

[11] Machida N, Taniguchi T, Nakamura T, Kiryu K. Cardio-histopathological observations on aborted equine fetuses infected with equid herpesvirus 1 (EHV-1). J Comp Pathol. 1997;116:379-85.

[12] Rodriguez HO, Guerrero NA, Fortes A, Santi-Rocca J, Girones N, Fresno M. Trypanosoma cruzi strains cause different myocarditis patterns in infected mice. Acta Trop. 2014;139:57-66.

[13] Chen S, Kapturczak M, Loiler SA, Zolotukhin S, Glushakova OY, Madsen KM, et al. Efficient transduction of vascular endothelial cells with recombinant adeno-associated virus serotype 1 and 5 vectors. Hum Gene Ther. 2005;16:235-47.

[14] Teramoto S, Ishii T, Matsuse T, Fukuchi Y. Recombinant adeno-associated virus vectors efficiently transduce foreign gene into bovine aortic endothelial cells: comparison with adenovirus vectors. Jpn J Pharmacol. 2000;84:206-12.

[15] Friedman HM, Macarak EJ, MacGregor RR, Wolfe J, Kefalides NA. Virus infection of endothelial cells. J Infect Dis. 1981;143:266-73.

[16] Chang CH, Huang Y, Issekutz AC, Griffith M, Lin KH, Anderson R. Interleukin-1alpha released from epithelial cells after adenovirus type 37 infection activates intercellular adhesion molecule 1 expression on human vascular endothelial cells. J Virol. 2002;76:427-31.

[17] Rolling F, Nong Z, Pisvin S, Collen D. Adeno-associated virus-mediated gene transfer into rat carotid arteries. Gene Ther. 1997;4:757-61.

[18] Visseren FL, Bouwman JJ, Bouter KP, Diepersloot RJ, de Groot PH, Erkelens DW. Procoagulant activity of endothelial cells after infection with respiratory viruses. Thromb Haemost. 2000;84:319-24.

[19] Tsai ML, Chen SL, Chou PI, Wen LY, Tsai RJ, Tsao YP. Inducible adeno-associated virus vector-delivered transgene expression in corneal endothelium. Invest Ophthalmol Vis Sci. 2002;43:751-7.

[20] Dropulic B, Masters CL. Entry of neurotropic arboviruses into the central nervous system: an in vitro study using mouse brain endothelium. J Infect Dis. 1990;161:685-91.

[21] Lander HM, Grant AM, Albrecht T, Hill T, Peters CJ. Endothelial cell permeability and adherens junction disruption induced by junin virus infection. Am J Trop Med Hyg. 2014;90:993-1002.

[22] Lukashevich IS, Maryankova R, Vladyko AS, Nashkevich N, Koleda S, Djavani M, et al. Lassa and Mopeia virus replication in human monocytes/macrophages and in endothelial cells: different effects on IL-8 and TNF-alpha gene expression. J Med Virol. 1999;59:552-60.

[23] Gomez RM, Pozner RG, Lazzari MA, D'Atri LP, Negrotto S, Chudzinski-Tavassi AM, et al. Endothelial cell function alteration after Junin virus infection. Thromb Haemost. 2003;90:326-33.

[24] Rudd PA, Wilson J, Gardner J, Larcher T, Babarit C, Le TT, et al. Interferon response factors 3 and 7 protect against Chikungunya virus hemorrhagic fever and shock. J Virol. 2012;86:9888-98.

[25] Friedman HM, Wolfe J, Kefalides NA, Macarak EJ. Susceptibility of endothelial cells derived from different blood vessels to common viruses. In Vitro Cell Dev Biol. 1986;22:397-401.

[26] Coyne CB, Bozym R, Morosky SA, Hanna SL, Mukherjee A, Tudor M, et al. Comparative RNAi screening reveals host factors involved in enterovirus infection of polarized endothelial monolayers. Cell Host Microbe. 2011;9:70-82.

[27] Zanone MM, Favaro E, Ferioli E, Huang GC, Klein NJ, Perin PC, et al. Human pancreatic islet endothelial cells express coxsackievirus and adenovirus receptor and are activated by coxsackie B virus infection. FASEB J. 2007;21:3308-17.

[28] Kuhnl A, Rien C, Spengler K, Kryeziu N, Sauerbrei A, Heller R, et al. Characterization of coxsackievirus B3 replication in human umbilical vein endothelial cells. Med Microbiol Immunol. 2014;203:217-29.

[29] Saijets S, Ylipaasto P, Vaarala O, Hovi T, Roivainen M. Enterovirus infection and activation of human umbilical vein endothelial cells. J Med Virol. 2003;70:430-9.

[30] Conaldi PG, Serra C, Mossa A, Falcone V, Basolo F, Camussi G, et al. Persistent infection of human vascular endothelial cells by group B coxsackieviruses. J Infect Dis. 1997;175:693-6.

[31] Vincent T, Pettersson RF, Crystal RG, Leopold PL. Cytokine-mediated downregulation of coxsackievirus-adenovirus receptor in endothelial cells. J Virol. 2004;78:8047-58.

[32] Funke C, Farr M, Werner B, Dittmann S, Uberla K, Piper C, et al. Antiviral effect of Bosentan and Valsartan during coxsackievirus B3 infection of human endothelial cells. J Gen Virol. 2010;91:1959-70.

[33] Nerheim PL, Meier JL, Vasef MA, Li WG, Hu L, Rice JB, et al. Enhanced cytomegalovirus infection in atherosclerotic human blood vessels. Am J Pathol. 2004;164:589-600.

[34] Ricotta D, Alessandri G, Pollara C, Fiorentini S, Favilli F, Tosetti M, et al. Adult human heart microvascular endothelial cells are permissive for non-lytic infection by human cytomegalovirus. Cardiovasc Res. 2001;49:440-8.

[35] Millard AL, Haberli L, Sinzger C, Ghielmetti M, Schneider MK, Bossart W, et al. Efficiency of porcine endothelial cell infection with human cytomegalovirus depends on both virus tropism and endothelial cell vascular origin. Xenotransplantation. 2010;17:274-87.

[36] Pampou S, Gnedoy SN, Bystrevskaya VB, Smirnov VN, Chazov EI, Melnick JL, et al. Cytomegalovirus genome and the immediate-early antigen in cells of different layers of human aorta. Virchows Arch. 2000;436:539-52.

[37] Fish KN, Soderberg-Naucler C, Mills LK, Stenglein S, Nelson JA. Human cytomegalovirus persistently infects aortic endothelial cells. J Virol. 1998;72:5661-8.

[38] Kahl M, Siegel-Axel D, Stenglein S, Jahn G, Sinzger C. Efficient lytic infection of human arterial endothelial cells by human cytomegalovirus strains. J Virol. 2000;74:7628-35.

[39] Hosogai M, Shima N, Nakatani Y, Inoue T, Iso T, Yokoo H, et al. Analysis of human cytomegalovirus replication in primary cultured human corneal endothelial cells. Br J Ophthalmol. 2015;99:1583-90.

[40] Bruns T, Zimmermann HW, Pachnio A, Li KK, Trivedi PJ, Reynolds G, et al. CMV infection of human sinusoidal endothelium regulates hepatic T cell recruitment and activation. J Hepatol. 2015;63:38-49.

[41] Luganini A, Cavaletto N, Raimondo S, Geuna S, Gribaudo G. Loss of the Human Cytomegalovirus US16 Protein Abrogates Virus Entry into Endothelial and Epithelial Cells by Reducing the Virion Content of the Pentamer. J Virol. 2017.

[42] Mazure G, Grundy JE, Nygard G, Hudson M, Khan K, Srai K, et al. Measles virus induction of human endothelial cell tissue factor procoagulant activity in vitro. J Gen Virol. 1994;75 ( Pt 11):2863-71.

[43] Woodroffe SB, Garnett HM, Danis VA. Interleukin-1 production and cell-activation response to cytomegalovirus infection of vascular endothelial cells. Arch Virol. 1993;133:295-308.

[44] Yaiw KC, Mohammad AA, Costa H, Taher C, Badrnya S, Assinger A, et al. Human Cytomegalovirus Up-Regulates Endothelin Receptor Type B: Implication for Vasculopathies? Open Forum Infect Dis. 2015;2:ofv155.

[45] Gustafsson RK, Jeffery HC, Yaiw KC, Wilhelmi V, Kostopoulou ON, Davoudi B, et al. Direct infection of primary endothelial cells with human cytomegalovirus prevents angiogenesis and migration. J Gen Virol. 2015;96:3598-612.

[46] Shen J, SS TT, Schrieber L, King NJ. Early E-selectin, VCAM-1, ICAM-1, and late major histocompatibility complex antigen induction on human endothelial cells by flavivirus and comodulation of adhesion molecule expression by immune cytokines. J Virol. 1997;71:9323-32.

[47] Dengler TJ, Raftery MJ, Werle M, Zimmermann R, Schonrich G. Cytomegalovirus infection of vascular cells induces expression of pro-inflammatory adhesion molecules by paracrine action of secreted interleukin-1beta. Transplantation. 2000;69:1160-8.

[48] Zamudio-Meza H, Castillo-Alvarez A, Gonzalez-Bonilla C, Meza I. Cross-talk between Rac1 and Cdc42 GTPases regulates formation of filopodia required for dengue virus type-2 entry into HMEC-1 cells. J Gen Virol. 2009;90:2902-11.

[49] Vervaeke P, Alen M, Noppen S, Schols D, Oreste P, Liekens S. Sulfated Escherichia coli K5 polysaccharide derivatives inhibit dengue virus infection of human microvascular endothelial cells by interacting with the viral envelope protein E domain III. PLoS One. 2013;8:e74035.

[50] Zhang JL, Wang JL, Gao N, Chen ZT, Tian YP, An J. Up-regulated expression of beta3 integrin induced by dengue virus serotype 2 infection associated with virus entry into human dermal microvascular endothelial cells. Biochem Biophys Res Commun. 2007;356:763-8.

[51] Velandia-Romero ML, Calderon-Pelaez MA, Castellanos JE. In Vitro Infection with Dengue Virus Induces Changes in the Structure and Function of the Mouse Brain Endothelium. PLoS One. 2016;11:e0157786.

[52] Azizan A, Sweat J, Espino C, Gemmer J, Stark L, Kazanis D. Differential proinflammatory and angiogenesis-specific cytokine production in human pulmonary endothelial cells, HPMEC-ST1.6R infected with dengue-2 and dengue-3 virus. J Virol Methods. 2006;138:211-7.

[53] Dalrymple N, Mackow ER. Productive dengue virus infection of human endothelial cells is directed by heparan sulfate-containing proteoglycan receptors. J Virol. 2011;85:9478-85.

[54] Qi Y, Li Y, Zhang Y, Zhang L, Wang Z, Zhang X, et al. IFI6 Inhibits Apoptosis via Mitochondrial-Dependent Pathway in Dengue Virus 2 Infected Vascular Endothelial Cells. PLoS One. 2015;10:e0132743.

[55] Kanlaya R, Pattanakitsakul SN, Sinchaikul S, Chen ST, Thongboonkerd V. The ubiquitin-proteasome pathway is important for dengue virus infection in primary human endothelial cells. J Proteome Res. 2010;9:4960-71.

[56] Arevalo MT, Simpson-Haidaris PJ, Kou Z, Schlesinger JJ, Jin X. Primary human endothelial cells support direct but not antibody-dependent enhancement of dengue viral infection. J Med Virol. 2009;81:519-28.

[57] Huber SA. VCAM-1 is a receptor for encephalomyocarditis virus on murine vascular endothelial cells. J Virol. 1994;68:3453-8.

[58] Zurbriggen A, Fujinami RS. Theiler's virus infection in nude mice: viral RNA in vascular endothelial cells. J Virol. 1988;62:3589-96.

[59] Takeda M, Miura R, Shiota K, Hirasawa K, Lee MJ, Itagaki SI, et al. Distribution of viral RNA in the spinal cord of DBA/2 mice developing biphasic paralysis following infection with the D variant of encephalomyocarditis virus (EMC-D). Int J Exp Pathol. 1995;76:441-7.

[60] Xiong A, Clarke-Katzenberg RH, Valenzuela G, Izumi KM, Millan MT. Epstein-Barr virus latent membrane protein 1 activates nuclear factor-kappa B in human endothelial cells and inhibits apoptosis. Transplantation. 2004;78:41-9.

[61] Jones K, Rivera C, Sgadari C, Franklin J, Max EE, Bhatia K, et al. Infection of human endothelial cells with Epstein-Barr virus. J Exp Med. 1995;182:1213-21.

[62] Bahr U, Muranyi W, Muller S, Kehm R, Handermann M, Darai G, et al. Bovine aortic endothelial cells are susceptible to Hantaan virus infection. Virology. 2004;321:1-7.

[63] Pensiero MN, Sharefkin JB, Dieffenbach CW, Hay J. Hantaan virus infection of human endothelial cells. J Virol. 1992;66:5929-36.

[64] McNulty S, Flint M, Nichol ST, Spiropoulou CF. Host mTORC1 signaling regulates andes virus replication. J Virol. 2013;87:912-22.

[65] Gorbunova EE, Gavrilovskaya IN, Mackow ER. Slit2-Robo4 receptor responses inhibit ANDV directed permeability of human lung microvascular endothelial cells. Antiviral Res. 2013;99:108-12.

[66] Kraus AA, Raftery MJ, Giese T, Ulrich R, Zawatzky R, Hippenstiel S, et al. Differential antiviral response of endothelial cells after infection with pathogenic and nonpathogenic hantaviruses. J Virol. 2004;78:6143-50.

[67] Kim IW, Hwang JY, Kim SK, Kim JK, Park HS. Interferon-stimulated genes response in endothelial cells following Hantaan virus infection. J Korean Med Sci. 2007;22:987-92.

[68] Taylor SL, Wahl-Jensen V, Copeland AM, Jahrling PB, Schmaljohn CS. Endothelial cell permeability during hantavirus infection involves factor XII-dependent increased activation of the kallikrein-kinin system. PLoS Pathog. 2013;9:e1003470.

[69] Shin OS, Yanagihara R, Song JW. Distinct innate immune responses in human macrophages and endothelial cells infected with shrew-borne hantaviruses. Virology. 2012;434:43-9.

[70] Pircher J, Czermak T, Merkle M, Mannell H, Krotz F, Ribeiro A, et al. Hepatitis C virus induced endothelial inflammatory response depends on the functional expression of TNFalpha receptor subtype 2. PLoS One. 2014;9:e113351.

[71] Fletcher NF, Wilson GK, Murray J, Hu K, Lewis A, Reynolds GM, et al. Hepatitis C virus infects the endothelial cells of the blood-brain barrier. Gastroenterology. 2012;142:634-43 e6.

[72] Balasubramanian A, Munshi N, Koziel MJ, Hu Z, Liang TJ, Groopman JE, et al. Structural proteins of Hepatitis C virus induce interleukin 8 production and apoptosis in human endothelial cells. J Gen Virol. 2005;86:3291-301.

[73] Blum P, Pircher J, Merkle M, Czermak T, Ribeiro A, Mannell H, et al. Arterial thrombosis in the context of HCV-associated vascular disease can be prevented by protein C. Cell Mol Immunol. 2016.

[74] Rodriguez-Inigo E, Casqueiro M, Bartolome J, Barat A, Caramelo C, Ortiz A, et al. Hepatitis C virus RNA in kidney biopsies from infected patients with renal diseases. J Viral Hepat. 2000;7:23-9.

[75] Caruso A, Favilli F, Rotola A, Comar M, Horejsh D, Alessandri G, et al. Human herpesvirus-6 modulates RANTES production in primary human endothelial cell cultures. J Med Virol. 2003;70:451-8.

[76] Rotola A, Di Luca D, Cassai E, Ricotta D, Giulio A, Turano A, et al. Human herpesvirus 6 infects and replicates in aortic endothelium. J Clin Microbiol. 2000;38:3135-6.

[77] Huang W, Chen X, Li Q, Li P, Zhao G, Xu M, et al. Inhibition of intercellular adhesion in herpex simplex virus infection by glycyrrhizin. Cell Biochem Biophys. 2012;62:137-40.

[78] Haruki T, Miyazaki D, Inata K, Sasaki S, Yamamoto Y, Kandori M, et al. Indoleamine 2,3-dioxygenase 1 in corneal endothelial cells limits herpes simplex virus type 1-induced acquired immune response. Br J Ophthalmol. 2015;99:1435-42.

[79] Sugioka K, Drake JD, Fukuda M, Shimomura Y, Hwang DG. Susceptibility of human corneal endothelial cells to HSV-1 infection. Curr Eye Res. 2005;30:863-9.

[80] Takeda S, Miyazaki D, Sasaki S, Yamamoto Y, Terasaka Y, Yakura K, et al. Roles played by toll-like receptor-9 in corneal endothelial cells after herpes simplex virus type 1 infection. Invest Ophthalmol Vis Sci. 2011;52:6729-36.

[81] Kim YC, Bang D, Lee S, Lee KH. The effect of herpesvirus infection on the expression of cell adhesion molecules on cultured human dermal microvascular endothelial cells. J Dermatol Sci. 2000;24:38-47.

[82] Brankin B, Hart MN, Cosby SL, Fabry Z, Allen IV. Adhesion molecule expression and lymphocyte adhesion to cerebral endothelium: effects of measles virus and herpes simplex 1 virus. J Neuroimmunol. 1995;56:1-8.

[83] Scheglovitova ON, Romanov YA, Maksianina EV, Svintsitskaya VA, Pronin AG. Herpes simplex type I virus infected human vascular endothelial cells induce the production of anti-viral and proinflammatory factors by peripheral blood leukocytes in vitro. Russ J Immunol. 2002;7:115-22.

[84] Gerson SL, Friedman HM, Cines DB. Viral infection of vascular endothelial cells alters production of colony-stimulating activity. J Clin Invest. 1985;76:1382-90.

[85] Key NS, Vercellotti GM, Winkelmann JC, Moldow CF, Goodman JL, Esmon NL, et al. Infection of vascular endothelial cells with herpes simplex virus enhances tissue factor activity and reduces thrombomodulin expression. Proc Natl Acad Sci U S A. 1990;87:7095-9.

[86] Holbach LM, Asano N, Naumann GO. Infection of the corneal endothelium in herpes simplex keratitis. Am J Ophthalmol. 1998;126:592-4.

[87] Ueda T, Miyake Y, Imoto K, Hattori S, Miyake S, Ishizaki T, et al. Distribution of human herpesvirus 6 and varicella-zoster virus in organs of a fatal case with exanthem subitum and varicella. Acta Paediatr Jpn. 1996;38:590-5.

[88] Moses AV, Bloom FE, Pauza CD, Nelson JA. Human immunodeficiency virus infection of human brain capillary endothelial cells occurs via a CD4/galactosylceramide-independent mechanism. Proc Natl Acad Sci U S A. 1993;90:10474-8.

[89] Cenacchi G, Re MC, Preda P, Pasquinelli G, Furlini G, Apkarian RP, et al. Human immunodeficiency virus type-1 (HIV-1) infection of endothelial cells in vitro: a virological, ultrastructural and immuno-cytochemical approach. J Submicrosc Cytol Pathol. 1992;24:155-61.

[90] Corbeil J, Evans LA, McQueen PW, Vasak E, Edward PD, Richman DD, et al. Productive in vitro infection of human umbilical vein endothelial cells and three colon carcinoma cell lines with HIV-1. Immunol Cell Biol. 1995;73:140-5.

[91] Kwok HH, Poon PY, Fok SP, Ying-Kit Yue P, Mak NK, Chan MC, et al. Anti-inflammatory effects of indirubin derivatives on influenza A virus-infected human pulmonary microvascular endothelial cells. Sci Rep. 2016;6:18941.

[92] Ocana-Macchi M, Bel M, Guzylack-Piriou L, Ruggli N, Liniger M, McCullough KC, et al. Hemagglutinin-dependent tropism of H5N1 avian influenza virus for human endothelial cells. J Virol. 2009;83:12947-55.

[93] Sun X, Zeng H, Kumar A, Belser JA, Maines TR, Tumpey TM. Constitutively Expressed IFITM3 Protein in Human Endothelial Cells Poses an Early Infection Block to Human Influenza Viruses. J Virol. 2016;90:11157-67.

[94] Chan MC, Chan RW, Yu WC, Ho CC, Chui WH, Lo CK, et al. Influenza H5N1 virus infection of polarized human alveolar epithelial cells and lung microvascular endothelial cells. Respir Res. 2009;10:102.

[95] Wang W, Mu X, Zhao L, Wang J, Chu Y, Feng X, et al. Transcriptional response of human umbilical vein endothelial cell to H9N2 influenza virus infection. Virology. 2015;482:117-27.

[96] Sumikoshi M, Hashimoto K, Kawasaki Y, Sakuma H, Suzutani T, Suzuki H, et al. Human influenza virus infection and apoptosis induction in human vascular endothelial cells. J Med Virol. 2008;80:1072-8.

[97] Davis LE, Kornfeld M, Daniels RS, Skehel JJ. Experimental influenza causes a non-permissive viral infection of brain, liver and muscle. J Neurovirol. 2000;6:529-36.

[98] Abdullah H, Brankin B, Brady C, Cosby SL. Wild-type measles virus infection upregulates poliovirus receptor-related 4 and causes apoptosis in brain endothelial cells by induction of tumor necrosis factor-related apoptosis-inducing ligand. J Neuropathol Exp Neurol. 2013;72:681-96.

[99] Andres O, Obojes K, Kim KS, ter Meulen V, Schneider-Schaulies J. CD46- and CD150-independent endothelial cell infection with wild-type measles viruses. J Gen Virol. 2003;84:1189-97.

[100] Cosby SL, Brankin B. Measles virus infection of cerebral endothelial cells and effect on their adhesive properties. Vet Microbiol. 1995;44:135-9.

[101] Isaacson SH, Asher DM, Godec MS, Gibbs CJ, Jr., Gajdusek DC. Widespread, restricted low-level measles virus infection of brain in a case of subacute sclerosing panencephalitis. Acta Neuropathol. 1996;91:135-9.

[102] Esolen LM, Takahashi K, Johnson RT, Vaisberg A, Moench TR, Wesselingh SL, et al. Brain endothelial cell infection in children with acute fatal measles. J Clin Invest. 1995;96:2478-81.

[103] Schmidt-Lucke C, Spillmann F, Bock T, Kuhl U, Van Linthout S, Schultheiss HP, et al. Interferon beta modulates endothelial damage in patients with cardiac persistence of human parvovirus b19 infection. J Infect Dis. 2010;201:936-45.

[104] Duechting A, Tschope C, Kaiser H, Lamkemeyer T, Tanaka N, Aberle S, et al. Human parvovirus B19 NS1 protein modulates inflammatory signaling by activation of STAT3/PIAS3 in human endothelial cells. J Virol. 2008;82:7942-52.

[105] von Kietzell K, Pozzuto T, Heilbronn R, Grossl T, Fechner H, Weger S. Antibody-mediated enhancement of parvovirus B19 uptake into endothelial cells mediated by a receptor for complement factor C1q. J Virol. 2014;88:8102-15.

[106] Pasquinelli G, Bonvicini F, Foroni L, Salfi N, Gallinella G. Placental endothelial cells can be productively infected by Parvovirus B19. J Clin Virol. 2009;44:33-8.

[107] Tzang BS, Tsai CC, Chiu CC, Shi JY, Hsu TC. Up-regulation of adhesion molecule expression and induction of TNF-alpha on vascular endothelial cells by antibody against human parvovirus B19 VP1 unique region protein. Clin Chim Acta. 2008;395:77-83.

[108] Magro CM, Nuovo G, Ferri C, Crowson AN, Giuggioli D, Sebastiani M. Parvoviral infection of endothelial cells and stromal fibroblasts: a possible pathogenetic role in scleroderma. J Cutan Pathol. 2004;31:43-50.

[109] Dyrsen ME, Iwenofu OH, Nuovo G, Magro CM. Parvovirus B19-associated catastrophic endothelialitis with a Degos-like presentation. J Cutan Pathol. 2008;35 Suppl 1:20-5.

[110] Arnold R, Konig W. Respiratory syncytial virus infection of human lung endothelial cells enhances selectively intercellular adhesion molecule-1 expression. J Immunol. 2005;174:7359-67.

[111] Geyer H, Bauer M, Neumann J, Ludde A, Rennert P, Friedrich N, et al. Gene expression profiling of rubella virus infected primary endothelial cells of fetal and adult origin. Virol J. 2016;13:21.

[112] Perelygina L, Zheng Q, Metcalfe M, Icenogle J. Persistent infection of human fetal endothelial cells with rubella virus. PLoS One. 2013;8:e73014.

[113] Lazar M, Perelygina L, Martines R, Greer P, Paddock CD, Peltecu G, et al. Immunolocalization and Distribution of Rubella Antigen in Fatal Congenital Rubella Syndrome. EBioMedicine. 2016;3:86-92.

[114] Sharma J, Blase JR, Hoft DF, Marentette JO, Turk J, McHowat J. Mice with Genetic Deletion of Group VIA Phospholipase A2beta Exhibit Impaired Macrophage Function and Increased Parasite Load in Trypanosoma cruzi-Induced Myocarditis. Infect Immun. 2016;84:1137-42.

[115] Morris SA, Wittner M, Weiss L, Hatcher VB, Tanowitz HB, Bilezikian JP, et al. Extracellular matrix derived from Trypanosoma cruzi infected endothelial cells directs phenotypic expression. J Cell Physiol. 1990;145:340-6.

[116] Tanowitz HB, Gumprecht JP, Spurr D, Calderon TM, Ventura MC, Raventos-Suarez C, et al. Cytokine gene expression of endothelial cells infected with Trypanosoma cruzi. J Infect Dis. 1992;166:598-603.

[117] Todorov AG, Andrade D, Pesquero JB, Araujo Rde C, Bader M, Stewart J, et al. Trypanosoma cruzi induces edematogenic responses in mice and invades cardiomyocytes and endothelial cells in vitro by activating distinct kinin receptor (B1/B2) subtypes. FASEB J. 2003;17:73-5.

[118] Campos-Estrada C, Liempi A, Gonzalez-Herrera F, Lapier M, Kemmerling U, Pesce B, et al. Simvastatin and Benznidazole-Mediated Prevention of Trypanosoma cruzi-Induced Endothelial Activation: Role of 15-epi-lipoxin A4 in the Action of Simvastatin. PLoS Negl Trop Dis. 2015;9:e0003770.

[119] Huang H, Calderon TM, Berman JW, Braunstein VL, Weiss LM, Wittner M, et al. Infection of endothelial cells with Trypanosoma cruzi activates NF-kappaB and induces vascular adhesion molecule expression. Infect Immun. 1999;67:5434-40.

[120] Rokita H, Kupiec T, Guzik K, Koj A. Vaccinia virus-regulated acute phase cytokine production in human fibroblasts, U937 cells and endothelium. Mediators Inflamm. 1998;7:73-8.

[121] Nikkels AF, Debrus S, Sadzot-Delvaux C, Piette J, Rentier B, Pierard GE. Localization of varicella-zoster virus nucleic acids and proteins in human skin. Neurology. 1995;45:S47-9.

[122] Nagel MA, Traktinskiy I, Azarkh Y, Kleinschmidt-DeMasters B, Hedley-Whyte T, Russman A, et al. Varicella zoster virus vasculopathy: analysis of virus-infected arteries. Neurology. 2011;77:364-70.

[123] Verma S, Lo Y, Chapagain M, Lum S, Kumar M, Gurjav U, et al. West Nile virus infection modulates human brain microvascular endothelial cells tight junction proteins and cell adhesion molecules: Transmigration across the in vitro blood-brain barrier. Virology. 2009;385:425-33.

[124] Roe K, Orillo B, Verma S. West Nile virus-induced cell adhesion molecules on human brain microvascular endothelial cells regulate leukocyte adhesion and modulate permeability of the in vitro blood-brain barrier model. PLoS One. 2014;9:e102598.

[125] Hussmann KL, Fredericksen BL. Differential induction of CCL5 by pathogenic and non-pathogenic strains of West Nile virus in brain endothelial cells and astrocytes. J Gen Virol. 2014;95:862-7.

[126] Hasebe R, Suzuki T, Makino Y, Igarashi M, Yamanouchi S, Maeda A, et al. Transcellular transport of West Nile virus-like particles across human endothelial cells depends on residues 156 and 159 of envelope protein. BMC Microbiol. 2010;10:165.

[127] Paddock CD, Nicholson WL, Bhatnagar J, Goldsmith CS, Greer PW, Hayes EB, et al. Fatal hemorrhagic fever caused by West Nile virus in the United States. Clin Infect Dis. 2006;42:1527-35.

[128] Khaiboullina SF, Rizvanov AA, Holbrook MR, St Jeor S. Yellow fever virus strains Asibi and 17D-204 infect human umbilical cord endothelial cells and induce novel changes in gene expression. Virology. 2005;342:167-76.
